# Intranasal esketamine + Intensive CBT: a 12 months follow-up of two complicated cases of Treatment Resistant Depression at high suicidal risk

**DOI:** 10.1192/j.eurpsy.2023.1792

**Published:** 2023-07-19

**Authors:** V. Martiadis, F. Raffone, R. Cerlino, S. Testa, M. Russo

**Affiliations:** Department of Mental Health, ASL Napoli 1 Centro, Napoli, Italy

## Abstract

**Introduction:**

Treatment Resistant Depression (TRD) is a complex, heterogeneous and multifactorial clinical condition that affects patients’ quality of life, their psychosocial functioning as well as suicidal risk. Intranasal esketamine is a new add-on treatment specifically approved for TRD.

**Objectives:**

The aim of the study was to evaluate the efficacy and safety of intranasal esketamine treatment combined with intensive Cognitive Behavioral psychotherapy (CBT), together with treatment satisfaction, in two complex clinical cases of TRD with high suicidal risk in a 12 months follow-up.

**Methods:**

Two male patients, 67 and 63 years old, with TRD, defined by at least two therapeutic failures with SSRI/SNRI and positive screening for high suicidal risk at the Columbia Suicide Severity Rating Scale, were selected for treatment with intranasal esketamine + CBT as an add-on to SSRI/SNRI antidepressant therapy. Psychopathological assessment were made by means of Hamilton Depression Rating Scale (HAM-D), Hamilton Anxiety Rating Scale (HAM-A), Columbia Suicide Severity Rating Scale (C-SSRS), Clinical Global Impression (CGI), Short Form Health Questionnaire (SF-36 items) at T0, every 7 days for the first 3 months, then every month. Treatment satisfaction was evaluated by means of the Client Satisfaction Questionnaire (CSQ-8), administered by trained nursing staff at 1, 3, 6 and 12 months. CBT specifically focused on depression was administered by a certified psychotherapist, weekly for the first 4 months, fortnightly for the next 3 months, monthly for the remaining 3 months.

**Results:**

After 2 administrations of esketamine the total HAM-D score was reduced by an average of 10 units and the suicidal risk was progressively reduced to zero according to C-SSRS. After 12 months one of the two patients reached and actually maintains clinical remission; the other one maintains a condition of mild depression; both without suicidal ideation and with a significant increase in perceived quality of life. Treatment was well tolerated, with mild and temporary adverse effects, self-limited to the administration sessions. CBT has contributed to increasing insight, cognitive resources, social interaction and self-esteem, and has made it possible to structure and carry on new life projects. The variation of the mean scores for CSQ-8 shows that esketamine + CBT treatment was considered as very satisfactory throughout the observation period.

**Conclusions:**

Intranasal esketamine associated with intensive CBT sessions showed to be effective, safe and satisfactory in the real world clinical management of two complex cases of TRD with high suicidal risk, improving quality of life, social functioning and eliminating suicidal ideation within 12 months follow-up. Satisfaction with the treatment contributed to strengthening adherence and improving the operator-patient therapeutic relationship.

**Disclosure of Interest:**

None Declared

